# Supplementary material for: The Effects of Prenatal Supplementation with β-Hydroxy-β-Methylbutyrate and/or Alpha-Ketoglutaric Acid on the Development and Maturation of Mink Intestines Are Dependent on the Number of Pregnancies and the Sex of the Offspring
Source: Animals (Basel). 2021 May 20;11(5):1468. doi: 10.3390/ani11051468 (PMC8160670; doi:10.3390/ani11051468)
Supplement: Supplementary file 1 [file animals-11-01468-s001.zip › animals-1186307-supplementary.pdf]

Table S1. The effect of the main factors (type of mother – primiparous or multiparous), diet/treatment and offspring sex) and their interactions on histomorphometric parameters of the duodenum of male and female offspring born from primiparous and multiparous dams supplemented with HMB and/or AKG during the gestational period.

P value for factors and interactions	Mother	Diet	Sex	Mother *Diet	Mother *Sex	Diet*Sex	Mother *Diet*Sex	C*HMB	C*AKG	C*HMB+AKG	HMB*AKG	HMB*HMB+AKG	AKG*HMB+AKG
**Thickness of the mucosa (μm)**	0.228	0.235	0.146	0.000	0.000	0.000	0.018	0.726	0.977	0.770	0.470	0.186	0.944
**Thickness of the submucosa (μm)**	0.000	0.063	0.833	0.000	0.189	0.004	0.070	0.648	0.294	0.040	0.935	0.445	0.806
**Thickness of the inner muscle layer (μm)**	0.005	0.000	0.000	0.116	0.000	0.001	0.091	0.095	0.967	0.005	0.028	0.745	0.001
**Thickness of the outer muscle layer (μm)**	0.000	0.040	0.000	0.000	0.000	0.000	0.002	0.357	0.599	0.969	0.021	0.637	0.325
**Total thickness of muscularis (μm)**	0.000	0.025	0.000	0.000	0.000	0.000	0.003	0.218	0.733	0.795	0.017	0.747	0.209
**Muscle to mucosa ratio**	0.316	0.000	0.000	0.000	0.000	0.000	0.000	0.777	0.003	0.851	0.000	0.294	0.040
**Submucosa to mucosa ratio**	0.006	0.806	0.161	0.000	0.043	0.001	0.000	0.989	1.000	0.923	0.995	0.778	0.897
**Total number of crypts/mm**	0.900	0.142	0.045	0.004	0.343	0.977	0.002	0.319	0.135	0.841	0.969	0.815	0.543
**Number of open crypts/mm**	0.057	0.000	0.003	0.002	0.000	0.094	0.308	0.488	0.134	0.322	0.002	0.992	0.001

**Number of closed crypts/mm**	0.031	0.000	0.077	0.000	0.000	0.029	0.017	0.000	1.000	0.025	0.000	0.422	0.033
**Number of undamaged villi/mm**	0.000	0.005	0.867	0.122	0.312	0.003	0.015	0.021	1.000	0.101	0.027	0.937	0.122
**Number of damaged villi/mm**	0.000	0.004	0.000	0.010	0.000	0.000	0.016	0.218	0.003	0.015	0.433	0.711	0.971
**Total number of villi/mm**	0.000	0.001	0.050	0.006	0.036	0.006	0.024	0.004	0.521	0.001	0.181	0.987	0.087
**Number of crypts to villi/mm ratio**	0.000	0.000	0.000	0.000	0.002	0.000	0.001	0.000	0.018	0.002	0.133	0.427	0.917
**Number of enterocytes/mm**	0.001	0.005	0.062	0.000	0.060	0.001	0.982	0.992	0.964	0.016	0.870	0.006	0.062
**Number of Goblet cells/mm**	0.632	0.010	0.000	0.000	0.392	0.000	0.000	0.075	0.812	0.929	0.005	0.273	0.440
**Enterocytes to Goblet cells ratio**	0.186	0.025	0.010	0.000	0.828	0.000	0.039	0.055	0.997	0.978	0.031	0.142	0.930
**The height of enterocytes (µm)**	0.119	0.005	0.000	0.000	0.867	0.394	0.009	0.898	0.004	0.853	0.036	1.000	0.049
**Villi height (µm)**	0.016	0.000	0.108	0.001	0.019	0.000	0.000	0.000	0.940	0.873	0.001	0.003	0.998
**Villi width (µm)**	0.241	0.461	0.103	0.000	0.260	0.307	0.074	0.397	0.717	0.891	0.956	0.829	0.986
**Crypts width (µm)**	0.481	0.011	0.001	0.001	0.244	0.750	0.050	0.092	0.934	0.306	0.018	0.935	0.092

**Crypts depth (μm)**	0.000	0.000	0.000	0.000	0.000	0.000	0.000	0.125	0.000	0.000	0.092	0.000	0.007
**Villus/crypt ratio**	0.000	0.000	0.000	0.000	0.000	0.101	0.000	0.000	0.236	0.007	0.085	0.738	0.538
**Absorption surface [μm²]**	0.050	0.000	0.374	0.046	0.393	0.000	0.000	0.000	0.375	0.316	0.017	0.024	1.000

C, control group; HMB, β-Hydroxy-β-methylbutyrate at a daily dose of 0.02 g/kg of body weight; AKG, alpha-ketoglutaric acid at a daily dose of 0.4 g/kg of body weight; N = 12 in each group; significance level at α<0.05

Table S2. The effect of the main factors (type of mother – primiparous or multiparous), diet/treatment and offspring sex) and their interactions on histomorphometric parameters of the jejunum of male and female offspring born from primiparous and multiparous dams supplemented with HMB and/or AKG during the gestational period.

P value for factors and interactions	Mother	Diet	Sex	Mother *Diet	Mother *Sex	Diet*Sex	Mother *Diet*Sex	C*HMB	C*AKG	C*HMB+AKG	HMB* AKG	HMB* HMB+ AKG	AKG* HMB+ AKG
**Thickness of the mucosa (μm)**	0.136	0.000	0.000	0.000	0.586	0.038	0.018	0.546	0.012	0.050	0.303	0.001	0.000
**Thickness of the submucosa (μm)**	0.008	0.000	0.660	0.418	0.153	0.427	0.000	0.012	0.221	0.476	0.000	0.372	0.004
**Thickness of the inner muscle layer (μm)**	0.194	0.000	0.077	0.131	0.000	0.000	0.000	0.007	1.000	0.000	0.007	0.011	0.000
**Thickness of the outer muscle layer (μm)**	0.011	0.001	0.844	0.010	0.698	0.000	0.891	0.659	0.050	0.715	0.001	1.000	0.002
**Total thickness of muscularis (μm)**	0.003	0.000	0.193	0.001	0.075	0.000	0.075	0.045	0.430	0.005	0.000	0.901	0.000
**Muscle to mucosa ratio**	0.040	0.016	0.012	0.014	0.198	0.000	0.888	0.147	0.768	0.729	0.011	0.699	0.187
**Submucosa to mucosa ratio**	0.000	0.000	0.066	0.001	0.639	0.697	0.001	0.016	0.820	0.530	0.001	0.000	0.963
**Total number of crypts/mm**	0.082	0.004	0.279	0.467	0.583	0.833	0.000	0.777	0.295	0.002	0.852	0.040	0.250
**Number of open crypts/mm**	0.000	0.000	0.011	0.129	0.006	0.044	0.563	0.009	0.000	0.012	0.150	1.000	0.123

**Number of closed crypts/mm**	0.811	0.002	0.965	0.589	0.162	0.049	0.000	0.165	0.297	0.561	0.990	0.004	0.012
**Number of undamaged villi/mm**	0.570	0.870	0.662	0.757	0.554	0.666	0.010	1.000	0.901	0.998	0.870	0.994	0.959
**Number of damaged villi/mm**	0.000	0.000	0.000	0.013	0.000	0.000	0.000	0.000	0.000	0.014	0.047	0.001	0.701
**Total number of villi/mm**	0.000	0.005	0.020	0.283	0.013	0.000	0.000	0.003	0.043	0.430	0.848	0.216	0.681
**Number of crypts to villi/mm ratio**	0.000	0.000	0.001	0.211	0.005	0.008	0.002	0.002	0.000	0.000	0.907	0.307	0.716
**Number of enterocytes/mm**	0.028	0.004	0.353	0.003	0.000	0.000	0.000	0.533	0.120	0.653	0.002	0.059	0.718
**Number of Goblet cells/mm**	0.260	0.001	0.689	0.240	0.553	0.012	0.006	0.003	1.000	0.130	0.002	0.555	0.116
**Enterocytes to Goblet cells ratio**	0.006	0.008	0.358	0.028	0.000	0.188	0.000	0.004	0.712	0.280	0.088	0.365	0.888
**The height of enterocytes (µm)**	0.339	0.056	0.042	0.000	0.200	0.443	0.001	0.974	0.610	0.049	0.855	0.138	0.531
**Villi height (µm)**	0.540	0.000	0.000	0.000	0.373	0.249	0.022	0.000	0.000	0.442	0.998	0.000	0.000
**Villi width (µm)**	0.000	0.000	0.000	0.010	0.002	0.000	0.000	0.007	0.000	0.251	0.393	0.513	0.017
**Crypts width (µm)**	0.546	0.000	0.005	0.000	0.043	0.003	0.000	0.008	0.728	0.014	0.000	0.000	0.199

**Crypts depth (μm)**	0.000	0.000	0.652	0.000	0.954	0.000	0.000	0.148	0.067	0.306	0.000	0.981	0.000
**Villus/crypt ratio**	0.000	0.001	0.035	0.000	0.848	0.000	0.000	0.014	0.998	0.775	0.023	0.000	0.676
**Absorption surface (μm²)**	0.760	0.000	0.000	0.000	0.309	0.007	0.000	0.002	0.000	0.003	0.174	0.999	0.133

C, control group; HMB, β-Hydroxy-β-methylbutyrate at a daily dose of 0.02 g/kg of body weight; AKG, alpha-ketoglutaric acid at a daily dose of 0.4 g/kg of body weight; N = 12 in each group; significance level at $\alpha<0.05$

Table S3. The effect of the main factors (type of mother – primiparous or multiparous), diet/treatment and offspring sex) and their interactions on histomorphometric parameters of the ileum of male and female offspring born from primiparous and multiparous dams supplemented with HMB and/or AKG during the gestational period.

P value for factors and interactions	Mother	Diet	Sex	Mother *Diet	Mother *Sex	Diet*Sex	Mother *Diet*Sex	C*HMB	C*AKG	C*HMB+AKG	HMB* AKG	HMB* HMB+ AKG	AKG* HMB+ AKG
**Thickness of the mucosa (μm)**	0.046	0.000	0.880	0.003	0.345	0.280	0.136	0.000	0.000	0.001	0.612	0.246	0.921
**Thickness of the submucosa (μm)**	0.000	0.000	0.058	0.002	0.651	0.029	0.006	0.000	0.310	0.002	0.051	0.909	0.231
**Thickness of the inner muscle layer (μm)**	0.001	0.000	0.516	0.038	0.000	0.000	0.036	0.040	0.019	0.496	0.000	0.000	0.439
**Thickness of the outer muscle layer (μm)**	0.044	0.000	0.879	0.000	0.067	0.002	0.042	0.001	0.994	0.952	0.000	0.006	0.862
**Total thickness of muscularis (μm)**	0.001	0.000	0.887	0.000	0.006	0.000	0.319	0.000	0.925	1.000	0.000	0.001	0.907
**Muscle to mucosa ratio**	0.000	0.002	0.622	0.012	0.046	0.000	0.012	0.957	0.004	0.063	0.020	0.199	0.789
**Submucosa to mucosa ratio**	0.000	0.000	0.491	0.000	0.275	0.001	0.001	0.000	0.005	0.000	0.012	0.841	0.116
**Total number of crypts/mm**	0.000	0.000	0.963	0.251	0.443	0.412	0.020	0.000	0.001	0.019	0.732	0.176	0.747
**Number of open crypts/mm**	0.000	0.000	0.186	0.000	0.003	0.057	0.000	0.000	0.021	0.000	0.049	0.883	0.254

**Number of closed crypts/mm**	0.457	0.022	0.060	0.000	0.012	0.215	0.013	0.828	0.980	0.059	0.599	0.351	0.020
**Number of undamaged villi/mm**	0.001	0.000	0.827	0.017	0.002	0.006	0.001	0.000	0.010	0.000	0.070	0.453	0.769
**Number of damaged villi/mm**	0.000	0.000	0.863	0.000	0.006	0.001	0.157	0.000	0.000	0.000	0.000	0.014	0.283
**Total number of villi/mm**	0.000	0.000	0.803	0.000	0.000	0.260	0.002	0.000	0.000	0.000	0.002	0.085	0.569
**Number of crypts to villi/mm ratio**	0.000	0.000	0.121	0.002	0.000	0.197	0.143	0.000	0.464	0.000	0.000	0.082	0.025
**Number of enterocytes/mm**	0.029	0.000	0.564	0.000	0.000	0.005	0.000	0.014	0.709	0.000	0.000	0.288	0.000
**Number of Goblet cells/mm**	0.417	0.000	0.859	0.017	0.073	0.000	0.223	0.002	0.010	0.562	0.949	0.000	0.000
**Enterocytes to Goblet cells ratio**	0.000	0.003	0.138	0.004	0.000	0.000	0.000	0.001	0.585	0.421	0.067	0.125	0.994
**The height of enterocytes (μm)**	0.166	0.722	0.501	0.017	0.123	0.108	0.027	0.868	0.841	1.000	1.000	0.854	0.826
**Villi height (μm)**	0.007	0.000	0.558	0.531	0.114	0.000	0.004	0.266	0.000	0.032	0.040	0.795	0.305
**Villi width (μm)**	0.302	0.008	0.186	0.000	0.119	0.000	0.000	0.534	0.869	0.068	0.149	0.688	0.007

**Crypts width (μm)**	0.000	0.000	0.049	0.034	0.000	0.001	0.003	0.000	0.861	0.910	0.002	0.000	0.468
**Crypts depth (μm)**	0.000	0.000	0.001	0.000	0.000	0.000	0.000	0.000	0.054	0.000	0.000	0.088	0.000
**Villus/crypt ratio**	0.000	0.000	0.118	0.000	0.001	0.017	0.109	0.000	0.023	0.000	0.000	0.990	0.001
**Absorption surface [μm²]**	0.428	0.000	0.611	0.070	0.506	0.000	0.030	0.996	0.000	0.020	0.001	0.038	0.628

C, control group; HMB, β-Hydroxy-β-methylbutyrate at a daily dose of 0.02 g/kg of body weight; AKG, alpha-ketoglutaric acid at a daily dose of 0.4 g/kg of body weight; N = 12 in each group; significance level at $\alpha<0.05$

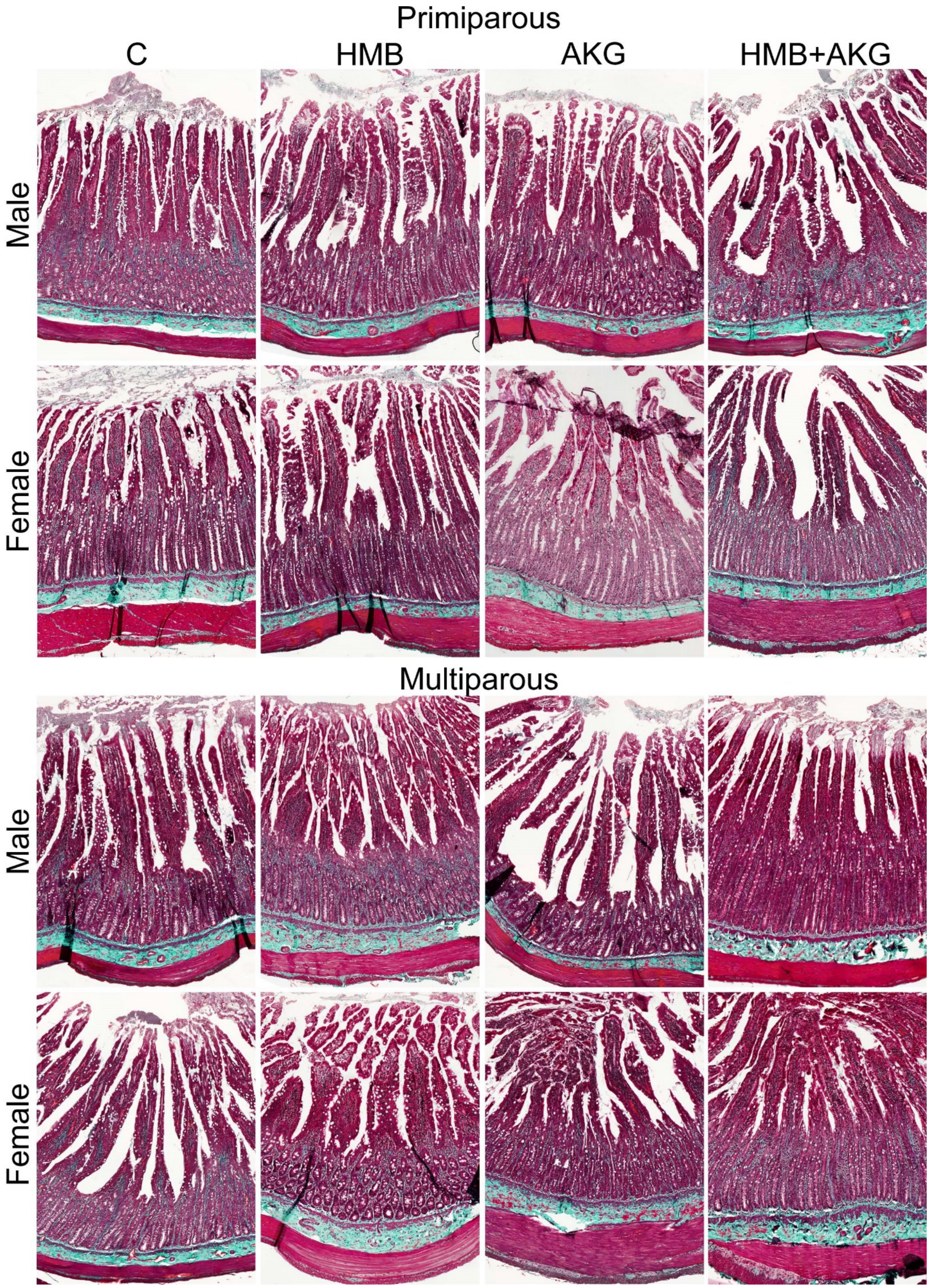

Figure 1S. A summary of Goldners trichrome staining images representative for jejunum. C, control group; HMB, β-Hydroxy-β-methylbutyrate at a daily dose of 0.02 g/kg of body weight; AKG, alpha-ketoglutaric acid at a daily dose of 0.4 g/kg of body weight; N = 12 in each group. Magnification x100.
